# Operational and organizational training for managing work stress among police investigation officers: a global perspective

**DOI:** 10.3389/fpsyg.2026.1919749

**Published:** 2026-09-25

**Authors:** Norazlina Zamberi, Zalmizy Hussin

**Affiliations:** School of Applied Psychology, Social Work & Policy, UUM College of Arts & Sciences, Universiti Utara Malaysia, Sintok, Malaysia

**Keywords:** job security, officer wellbeing, operational training, organizational intervention, police investigators, police occupational stress, professional identity, psychological capital

## Introduction

Criminal investigators face chronic demands beyond general police work: repeated analysis of traumatic digital evidence, heavy caseloads, cognitive overload, evidentiary scrutiny, and accountability for error (Gullon-Scott and Johnson, 2024; Schlosser et al., 2024; Strickland et al., 2023; Woodhams and Duran, 2024). Digitalization and rapidly changing technical requirements intensify these pressures while recruitment and retention problems restrict capacity (Drew et al., 2024). Burnout consequently threatens both officer wellbeing and investigative quality (Bezie et al., 2024).

The literature separates operational stressors, which are intrinsic to the work, from organizational stressors, which arise from how the institution is run, such as workload allocation, supervisory support, role clarity, and culture (Gavin and Porter, 2024). A finding that is decisive for training design is that organizational and operational stressors, rather than trauma exposure alone, predict most stress-related outcomes among investigative and forensic staff (Keech and Drew, 2026; Porter and Gavin, 2025). Among United States investigators exposed to CSAM, the frequency of exposure was not systematically related to mental health, apart from violent material, which was linked to elevated post-traumatic stress; agency-level factors such as control over workload, debriefing, and feedback on case outcomes were what mitigated harm (Mitchell et al., 2023).

This Opinion draws on peer-reviewed studies published between 2022 and 2026, identified through database searches on police and investigator occupational stress, psychological capital, technostress, and organizational interventions, prioritizing evidence specific to investigative and forensic roles and using the broader occupational-health literature where role-specific studies are scarce. On that basis, I argue that stress-management interventions for investigators should rest on two complementary pillars. Operational training strengthens the adaptive capacity of the individual officer; organizational training reshapes the leadership, climate, and work systems in which officers operate (Figure 1). Current practice still leans heavily toward the first pillar, locating both the problem and its remedy in the worker. I treat investigator resilience as a structurally enabled resource rather than a fixed personal trait.

**Figure 1:**
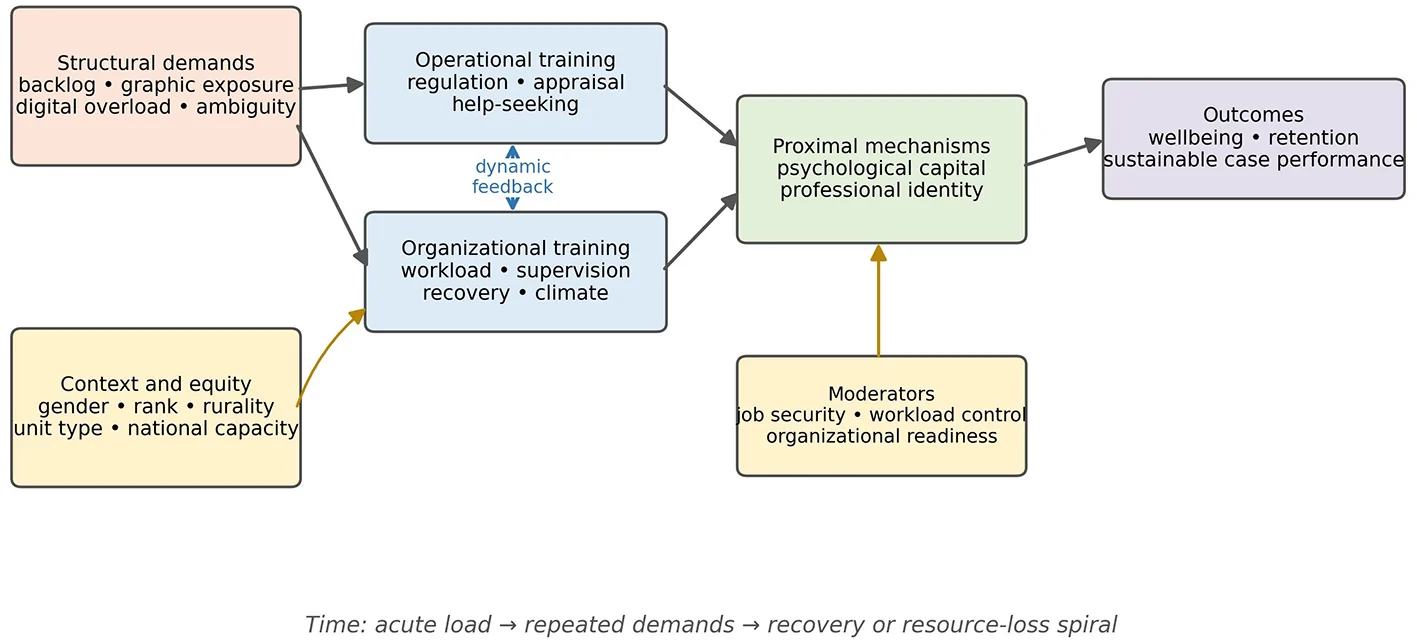
Integrative framework. Structural demands activate operational and organizational interventions; psychological capital and professional identity are proximal mechanisms; job security and context moderate effects; and wellbeing, retention, and sustainable case performance are outcomes.

## Investigative work as a structural condition

The evidence supports a structural rather than an episodic framing. Multidimensional assessments show that police stress tracks enduring features of the work environment, including job demands, control, support, role clarity, and organizational change, more than isolated critical incidents (Strukcinskiene et al., 2025). Within investigative and forensic units, organizational and operational demands, not trauma exposure alone, predict the largest share of stress-related outcomes, while supervisor support, peer support, and a positive psychosocial safety climate act as the main buffering resources (Keech and Drew, 2026). This pattern recasts exposure as one demand among several rather than the sole cause of harm (Drew et al., 2024).

The job demands-resources (JD-R) model explains how demands deplete wellbeing unless balanced by resources. Conservation of resources (COR) theory explains how continued depletion without recovery creates loss spirals and burnout (Demerouti et al., 2001; Hobfoll, 1989; Zhou et al., 2024). Operational training adds personal resources; organizational action protects them.

Integration requires a multilevel and temporal account. Structural demands such as backlog, traumatic exposure, digital overload and ambiguity first activate the JD-R health-impairment pathway. The two pillars then operate in a reciprocal cycle: operational training may improve immediate regulation, appraisal and help-seeking, while organizational training changes workload, supervisory behavior, recovery opportunities and psychological safety. These organizational changes determine whether personal gains are replenished or progressively depleted. COR theory therefore predicts that operational training is most useful during acute demand, whereas organizational redesign becomes increasingly important when demands are recurrent, controllable and system-generated. Professional identity is proposed as a mediator, not a presumed protection: stress may weaken identification through cynicism and perceived value conflict, while credible supervision and meaningful feedback may restore identification and its wellbeing benefits. Job security is proposed as a moderator because officers who fear transfer, stigma, career penalty or job loss may be less able to disclose strain or use support. The model predicts that combined interventions will be more durable when workload control, psychological safety and leadership accountability improve; it does not assume that combined interventions will always outperform individual training.

Digital transformation adds a further, persistent demand. Technostress, the strain from constant connectivity, information overload, and the intrusion of technology into recovery time, behaves as a chronic job demand associated with lower wellbeing and burnout, and it is intrinsic to investigative work built around digital evidence (Pansini et al., 2023; Mansuroglu and Smith, 2026). Two resources deserve emphasis because they locate where interventions should act. Professional identity mediates the link between occupational stress and wellbeing, and perceived job security moderates it, which indicates that meaning and stability are protective resources shaped largely by organizations rather than by individuals alone (Wu et al., 2025).

## The value and limits of operational training

Operational training targets the officer's own coping repertoire and has a credible evidence base. Mindfulness-based resilience training has shown feasibility and promising effects on stress in police samples (Christopher et al., 2024), and physiologically grounded programs that pair heart-rate-variability biofeedback with metacognitive skill practice are being trialed to build resilience in officers exposed to post-traumatic stress injuries (Andersen et al., 2023). These approaches develop psychological capital, the malleable resources of hope, efficacy, resilience, and optimism that are associated with lower burnout and turnover intention and with higher engagement and performance (Loghman et al., 2023).

The limits of a purely individual approach are equally clear, and they should be stated without overclaiming the alternative. Worker-directed interventions place the burden of adaptation on the person, can imply that distress signals personal weakness, and tend to fade once officers return to unchanged conditions. They do not remediate the organizational stressors that the investigator-specific evidence identifies as the deepest sources of harm (Gavin and Porter, 2024; Mitchell et al., 2023). Teaching investigators to regulate their physiology is useful, but it does not reduce an unmanageable caseload or a dismissive command climate.

## Organizational training and work-system redesign

Comparative reviews suggest that organizational and combined interventions may produce benefits comparable with, and sometimes more durable than, individual interventions in healthcare and general workplaces (Catapano et al., 2023; Dahlqvist et al., 2023). This evidence cannot be treated as direct proof for investigative policing. Its relevance is theoretical and conditional: reducing demands at source should protect recovery, but policing adds hierarchy, operational urgency, confidentiality requirements and occupational stigma. Accordingly, the present framework offers a testable proposition rather than an effectiveness claim. Organizational action should be staged according to readiness, fidelity, leadership commitment and resources, with individual training retained as an accessible component while system-level effects are evaluated.

For investigative units, this pillar includes trauma-informed supervision, case-outcome feedback, exposure and caseload review, breaks and recovery after graphic-material work, peer support, psychosocial safety monitoring, and leadership accountability (Mitchell et al., 2022, 2023; Keech and Drew, 2026). Leadership links the pillars because supportive leadership can strengthen psychological capital (Loghman et al., 2023). Table 1 distinguishes evidence from proposals requiring testing.

**Table 1:** Evidence-calibrated intervention matrix.

Intervention level	Core functions for investigative units	Evidence status	Boundary conditions/risks	Proposed evaluation indicators
Operational training	Mindfulness or resilience skills; physiological regulation; metacognitive appraisal; help-seeking and peer connection	Feasibility and promising effects reported in police samples; direct effects may be short-term	Useful during acute load; may shift responsibility to officers if system demands remain unchanged	Burnout, distress, secondary traumatic stress, skill use, help-seeking, adverse effects
Organizational training and redesign	Workload and exposure review; trauma-informed supervision; outcome feedback; protected recovery; psychosocial safety; leadership accountability	Strong systems rationale; direct investigative-policing trials remain limited	Requires readiness, leadership commitment, resources and confidentiality safeguards; may fail if imposed without trust	Reach, fidelity, workload control, climate, recovery time, absence, turnover intention
Combined, staged approach	Individual skill-building paired with demand reduction and supportive supervision; iterative adjustment over time	Plausible but not established as superior in investigative policing; transfer from other sectors is conditional	Most suitable when demands are recurrent and controllable; monitor burden, inequitable access and implementation drift	Cluster/stepped-wedge comparison; wellbeing, retention, case delay, rework and equity-stratified effects
Equity and access layer	Remote/regional access; protected time; independent referral; culturally appropriate providers; safeguards against career penalties	Implementation requirement and testable moderator, not an optional add-on	Gender, rank, rurality, unit type and national capacity may alter reach and effect	Uptake, perceived safety, access, workload relief and outcomes disaggregated by subgroup

Indicators are proposed evaluation measures unless directly tested in the cited studies.

Delivery should fit agency capacity. National laboratories may use exposure logging, specialist supervision, and rotation; small or rural forces may need regional case-sharing, remote consultation, peer supervision, and referral agreements. Internet-crime task forces may prioritize graphic-exposure limits and outcome feedback, while general detective units address backlog, autonomy, and recovery. In lower-resource settings, feasible minimums include workload review, supervisor training, confidential referral, and protected recovery. The functions are transferable, but implementation must reflect staffing, geography, labor protections, culture, and stigma.

Implementation barriers require active design responses. Hierarchy can be addressed through confidential reporting routes, supervisor accountability and protected escalation pathways; resource constraints through phased implementation and regional/shared services; stigma through routine, universal wellbeing processes rather than exceptional referral; confidentiality concerns through independent providers and clear data-governance rules; and competing priorities through integrating recovery, exposure review and case-outcome feedback into existing quality and supervision meetings. These strategies are proposed implementation mechanisms, not evidence of established effectiveness. Their value should be assessed using reach, fidelity, acceptability, equity of access and unintended-consequence indicators. Treating barriers as design conditions preserves the structural argument while acknowledging that organizational interventions can fail or impose new burdens if poorly implemented.

## Equity, access, and cross-national variation

Equity is a design requirement rather than a descriptive add-on. Access to resilience resources may vary by gender, rank, rurality, contract status, unit type and national capacity (Bezie et al., 2024; Porter and Gavin, 2025). Women officers may encounter additional caregiving demands, gendered expectations or lower psychological safety; junior officers may have less discretion to request recovery; rural officers may face distance, limited specialist services and confidentiality concerns in small teams. These conditions may alter both exposure to demands and the feasibility of each intervention. Implementation should therefore require disaggregated monitoring of reach, uptake, workload relief, perceived safety and outcomes by gender, rank, rurality and unit. Agencies should offer multiple access routes, remote and regional options, protected time, culturally appropriate providers and safeguards against career penalties. Core functions should be retained, but delivery should be adapted and tested rather than assumed to be equivalent across contexts.

## Discussion

The pillars are complementary but not interchangeable. Operational training can support functioning during acute load and may be particularly feasible when resources are limited. Organizational action determines whether those gains are maintained by changing recurring demands, recovery conditions and leadership climate. The framework specifies structural demands as antecedents; operational and organizational training as interacting interventions; psychological capital and professional identity as candidate mechanisms; job security, workload control, climate, gender, rank, rurality and national context as moderators or boundary conditions; and wellbeing, retention and sustainable case performance as outcomes. The evidentiary balance is uneven: individual interventions have more immediate feasibility evidence, whereas organizational redesign has a stronger systems rationale but fewer direct investigative trials. Recommendations should therefore be implemented as monitored, reversible learning cycles rather than universal prescriptions.

Research should test operational-only, organizational-only, and combined conditions using cluster-randomized or stepped-wedge designs over at least 12 months. Outcomes should cover burnout, secondary traumatic stress, distress, absence, retention, case delay, and rework. Tests should examine psychological capital, professional identity, workload control, support, and climate as mechanisms and compare effects by gender, rank, rurality, unit type, and national context. Null and adverse implementation effects should be reported because current investigator evidence remains largely cross-sectional or preliminary.

Policy should become auditable and equitable. Units should review caseload and graphic exposure regularly; provide confidential, trauma-informed supervision; record access to case-outcome feedback; protect recovery; and monitor climate, absence, turnover intention and support uptake. Universal exposure caps are pre-mature. Agencies should instead define local trigger points for mandatory workload review, document exceptions and test whether thresholds predict health and service outcomes. Senior leaders should be accountable for system indicators alongside case throughput. Every implementation should report reach and effects by gender, rank, rurality, unit type and national context, and should include safeguards against retaliation, disclosure of sensitive case information and unequal access. Where resources are limited, minimum standards should include workload review, trained supervision, confidential referral and protected recovery time, with regional partnerships used to extend specialist access.

The framework remains an evidence-informed working hypothesis. Investigator-specific studies identify plausible risks and protective practices, but causal evidence for organizational redesign is limited and evidence from other occupations is not automatically transferable. Agencies should therefore build officer skills, reduce recurrent resource depletion, and evaluate both workforce and investigative outcomes.
